# Artificial intelligence in the prediction of intraoperative red blood cell transfusion in cardiac surgery: a systematic review and diagnostic test accuracy meta-analysis

**DOI:** 10.1016/j.bjane.2026.844782

**Published:** 2026-06-25

**Authors:** Filipe Giordano Valério, Amanda Carneiro Rodrigues, Davi Ricardo Soares Gama de Amorim, Roberta Esterque Cantarino, Glauco Martins de Araujo, Jimmy Yusuf, Lecticia Vianna Leal Soares Bessa, Millena Mendonça Andrade Paes Leme, Marcella Freire de Campos Euzebio, Flávio Luiz Seixas, Alexandra Rezende Assad, Luis Antonio dos Santos Diego

**Affiliations:** a Universidade Federal Fluminense (UFF), Niterói, RJ, Brazil; b Faculdade de Ciências Médicas da Santa Casa de São Paulo, (FCMSCSP), São Paulo, SP, Brazil; c Universidade de Pernambuco (UPE), Recife, PE, Brazil; d Universidade Federal Fluminense, Institute of Computing, Niterói, RJ, Brazil; e Universidade Federal do Rio de Janeiro, Department of General and Specialized Surgery, Postgraduate Program in Surgical Sciences, Rio de Janeiro, RJ, Brazil; f Universidade Federal Fluminense (UFF), Faculdade de Medicina, Department of Surgery, Niterói, RJ, Brazil

**Keywords:** Algorithms, Artificial intelligence, Blood transfusion, Cardiac surgical procedures, Erythrocyte transfusion, Prediction algorithms

## Abstract

**Background:**

Red Blood Cell (RBC) transfusion in cardiac surgery is associated with risks. Conventional prediction scores lack accuracy, conflicting with Patient Blood Management (PBM) principles. Artificial Intelligence (AI) offers a potential avenue for developing precise, data-driven predictive models to enhance clinical decision-making and optimize patient outcomes.

**Methods:**

A systematic review and meta-analysis of diagnostic test accuracy studies was conducted following PRISMA guidelines, searching PubMed, Embase, and Cochrane Library databases until April 2025. Studies developing AI models to predict intraoperative Red Blood Cell (RBC) transfusions in adult cardiac surgery were included. Pooled sensitivity, specificity, and Area Under the receiver operating Characteristic Curve (AUC) were calculated using a bivariate random-effects model. To summarize overall diagnostic performance, Summary Receiver Operating Characteristic curves were generated.

**Results:**

Five studies encompassing 3,063 patients were analyzed in the meta-analysis. AI models demonstrated high pooled specificity, ranging from 86.3% (95% CI 82.8%‒89.1%) to 93.6% (95% CI 84.3%‒97.6%), whereas pooled sensitivity ranged from 50.3% (95% CI 20.9%‒79.6%) to 55.7% (95% CI 33.8%‒75.6%). AUC values ranged between 0.793 (95% CI 0.634‒0.899) and 0.892 (95% CI 0.740‒0.943). Preoperative hemoglobin levels and patient age were consistently identified as clinical predictors for intraoperative RBC transfusion.

**Conclusion:**

This systematic review and meta-analysis suggests that AI models may have potential for predicting intraoperative RBC transfusion in cardiac surgery, with consistently high specificity but only moderate sensitivity. However, given the low certainty of evidence, substantial heterogeneity, and reliance on non-standardized transfusion practices, these findings should be interpreted cautiously.

## Introduction

Intraoperative Red Blood Cell (RBC) transfusion is common in cardiac surgery, a context often complicated by hypovolemia and impaired tissue perfusion. However, transfusions carry risks, including infection, lung injury, transfusion-related immunomodulation, and increased morbidity and mortality.^1^, ^2^, ^3^, ^4^, ^5^, ^6^ Patient Blood Management (PBM) has emerged as a multimodal, evidence-based approach to optimize transfusion outcomes and enhance patient safety. PBM is structured around three pillars: 1) Optimizing red blood cell mass via screening, diagnosis, and treatment of anemia and iron deficiency; 2) Minimizing surgical and iatrogenic blood loss while managing bleeding and coagulopathies perioperatively; and 3) Optimizing physiological tolerance to anemia by improving oxygen delivery and reducing oxygen demand.^7^, ^8^, ^9^ This definition also underscores the need for shared decision-making, actively involving patients in the management of their own blood.

Despite increasing adoption of PBM principles, predicting intraoperative RBC transfusion in cardiac surgery remains challenging. Clinicians largely depend on general risk models, such as the STS score and EuroSCORE, which are designed to estimate mortality and major morbidity rather than hematological requirements.^10^^,^^11^ Although transfusion-specific tools, including “Transfusion Risk and Clinical Knowledge” (TRACK) and “Transfusion Risk Understanding Scoring Tool” (TRUST), have been developed to address this limitation, their predictive performance remains limited, resulting in reliance on clinical judgment and the team's prior experience.^2^^,^^12^ The absence of reliable prediction methods contributes to blood component overuse and increasing perioperative risk, in conflict with PBM principles.^2^^,^^4^^,^^13^

In this scenario, Artificial Intelligence (AI) emerges as a promising strategy to overcome these limitations and improve perioperative blood management. AI enables the analysis of high-dimensional clinical data to develop predictive models, with approaches varying in methodology and complexity.^1^^,^^4^^,^^13^ These approaches aim to support clinical decision-making, reduce unnecessary transfusions, mitigate patient risk, and optimize the use of blood products and hospital resources. Accordingly, this systematic review and meta-analysis evaluates the statistical robustness of different AI models for predicting intraoperative RBC transfusion in cardiac surgery.

## Methods

### Study design and protocol registration

This systematic review and meta-analysis was conducted in accordance with the guidelines of the Cochrane Collaboration^14^^,^^15^ and the Preferred Reporting Items for Systematic Reviews and Meta-Analyses (PRISMA) statement.^16^ The protocol was registered on September 16, 2025, before data extraction, in the International Prospective Register of Systematic reviews (PROSPERO) under registration CRD420251146515.

### Research question

The research question was elaborated using the PIT framework for diagnostic test accuracy meta-analysis, as recommended by the Cochrane Handbook for Systematic Reviews of Diagnostic Test Accuracy (v2.0): Population (P) ‒ patients undergoing cardiac surgery; Index test (I) ‒ use of AI algorithms; Target condition (T) ‒ Occurrence of RBC transfusion in the intraoperative period (described as “intraoperative” in four out of five articles, and “from the initiation of surgery until discharge to the intensive care unit” in Cunha et al.)^2^^,^^14^

### Data sources

Searches were systematically conducted in PubMed, Embase, and Cochrane Library, up to April 15, 2025.

### Search methods

The first author (F.V.) developed search strategies (Supplementary Table 1). A search of the reference lists of the studies included was conducted to ensure that no other relevant studies were overlooked.

### Eligibility criteria

This systematic review included studies evaluating the predictive accuracy of AI models for intraoperative RBC transfusion in adult patients undergoing cardiac surgery. Eligible studies reported the target population, applied AI-based index tests, and assessed intraoperative RBC transfusion prediction accuracy. Studies involving inappropriate populations, case reports, ongoing trials, conference abstracts, editorials, letters, comments, and studies without final results were also excluded.

### Study selection

**Figure 1:**
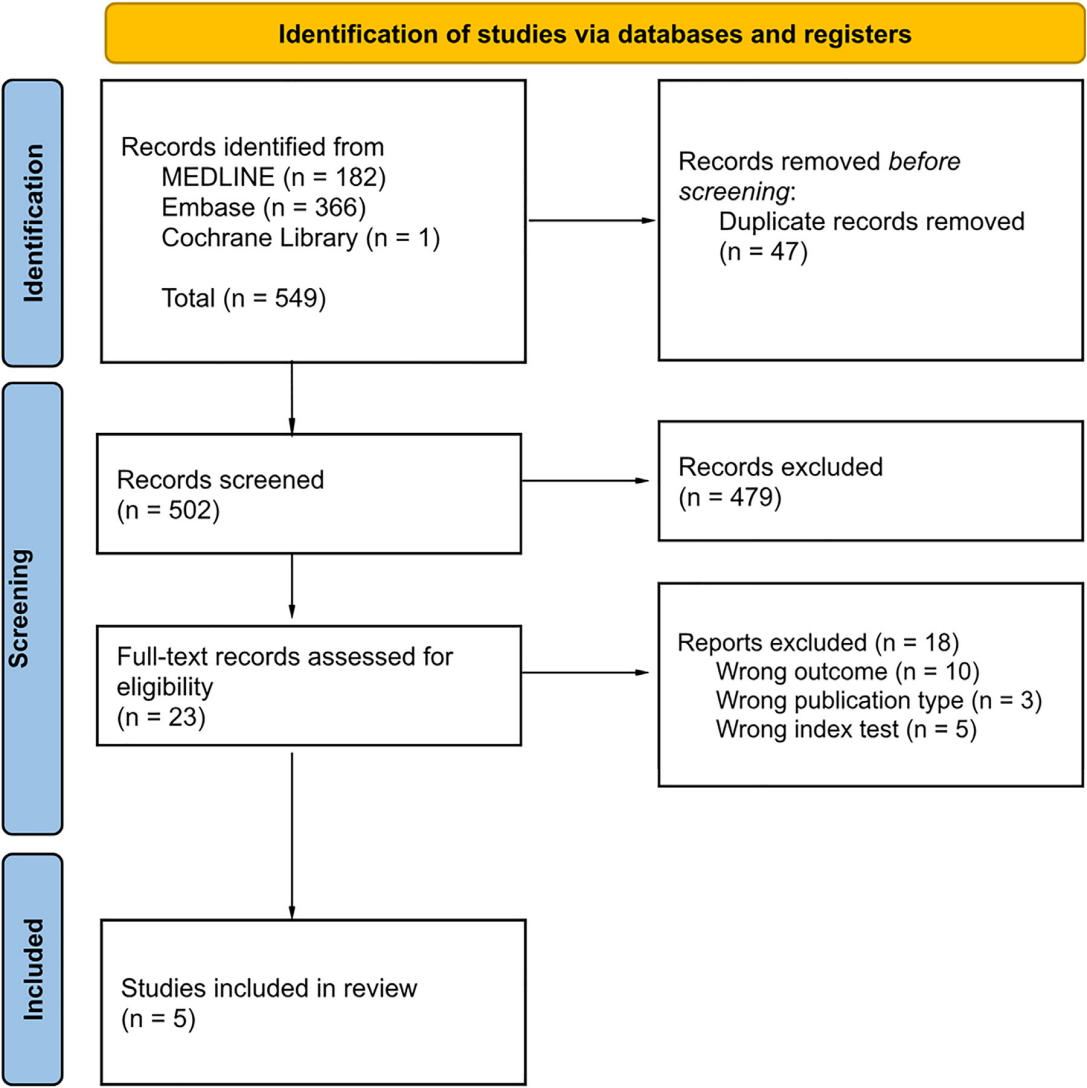
PRISMA flow diagram of study screening and selection.

Retrieved articles were imported into the Rayyan systematic review platform,^17^ deduplicated, and independently screened by three reviewers (F.V., L.B., J.Y.). Full texts were subsequently assessed when eligibility was unclear, and studies meeting the criteria were included (Fig. 1). Disagreements were resolved by a supervising reviewer (L.D.).

### Outcomes

Outcomes assessed in this review were the pooled sensitivity, specificity, and Area Under the Curve (AUC) for AI algorithms eligible for statistical analysis. All AI models assessed in two or more articles were included in this evaluation. To ensure stable pooled estimates and sufficient statistical power, models evaluated in four or more studies are described individually. Models assessed in fewer than four studies are summarized under “Other models” in the Results, with their Summary Receiver Operating Characteristic (SROC) curves and supplemental graphs provided in the Supplementary Material. Due to the limited sample sizes, results for these models should be interpreted with caution.

### Data extraction

Three blinded reviewers (J.Y., G.A., and A.R.) independently extracted data from each study, including authorship, year of publication, country, and patient characteristics (sample size per group, age, and sex), as well as predictors, AI models, type of surgery, hemoglobin, hematocrit, and diagnostic performance metrics (sensitivity, specificity, accuracy, and precision). Data were collected using Google Sheets and cross-checked for accuracy (Table 1). Corresponding authors were contacted by email to obtain missing data; no responses were received.

**Table 1:** Baseline characteristics of studies.

Study	Index tests	Types of surgery	Number of surgeries		Transfused patients	Female sex (%)	Age (years)	Hemoglobin (g/dL)	Hematocrit (%)	Red Blood Cells (10^12/L)	Platelets (10^9/L)	Prothrombin time (seconds)
			Training set	Testing set	n (%)	n (%)	Mean ± SD	Mean ± SD	Mean ± SD	Mean ± SD	Mean ± SD	Mean ± SD
**Sun, 2024**^6^	Extreme Gradient Boosting, Extreme Gradient Boosting, Extra Trees, Logistic Regression, Categorical Boosting, Adaptive Boosting, Linear Discriminant Analysis, Random Forest, Decision Tree, K Nearest Neighbor.	Minimally invasive direct coronary artery bypass (MIDCAB)	613	153	107 (13.97%)	221 (28.85%)	65.2 ± 9.5	13.1 ± 1.72	39.5 ± 5.1	4.28 ± 0.54	215.5 ± 56	12.91 ± 0.92
**Liu, 2021**^4^	Categorical Boosting, Light Gradient Boosting, Extreme Gradient Boosting, Extra Trees, Logistic Regression, Linear Discriminant Analysis, Random Forest, Adaptive Boosting, Naive Bayes, K Nearest Neighbor, Decision Tree, Quadratic Discriminant Analysis	Mitral and tricuspid valve surgery	473	204	166 (24.52%)	326 (48.15%)	57.3 ± 13.37	13.12 ± 1.82	39.36 ± 5.08	4.35 ± 0.6	186.50 ± 54.97	11.635 ± 0.97
**Chen, 2024**^3^	Logistic Regression, Categorical Boosting, Extra Trees, Naive Bayes (Gaussian and Bernoulli), Multilayer Perceptron, Adaptive Boosting, Random Forest, Light Gradient Boosting, Support Vector Machine, Extreme Gradient Boosting, Decision Tree	Surgical and transcatheter aortic valve replacement and implantation	491	211	267 (38.03%)	242 (34.47%)	55.76 ± 12.3	13.59 ± 1.81	41 ± 5	4.6 ± 0.6	169.19 ± 61.03	12.1 ± 3.45
**Cunha, 2023**^2^	Logistic Regression, Multilayer Perceptron, Random Forest, Support Vector Machine, TRACK, TRUST	Aortic surgery, valve surgery, coronary artery bypass grafting, combined surgery, and other cardiac surgeries (not specified)	396	99	284 (57.37%)	196 (39.6%)	56.66 ± 14.17	11.3 ± 2.17	33.9 ± 6.5	-	-	-
**Zhou, 2024**^1^	Extreme Gradient Boosting, Random Forest, Decision Tree, Categorical Boosting, Support Vector Machine, Logistic Regression	Valve replacement surgery	338	85	102 (24.11%)	199 (47.04%)	60.1 ± 11.4	13.65 ± 1.39	41.12 ± 4.73	-	172 ± 56.4	13.61 ± 4.68

TRACK, Transfusion Risk and Clinical Knowledge; TRUST, Transfusion Risk Understanding Scoring Tool.

### Quality assessment and risk of bias

The methodological quality of each study was verified using the PROBAST-AI^18^ tool by two blinded authors (R.C. and G.A.). This tool covers four domains, as follows: patients, predictors, outcome, and analysis. The first author (F.V.) resolved any discrepancies. Two authors (R.C., G.A.) independently assessed studies for quality and certainty of evidence in accordance with the Grading of Recommendations Assessment, Development and Evaluation (GRADE) tool.^19^

### Statistical analysis

As per the Cochrane Handbook for Systematic Reviews of Diagnostic Test Accuracy (v2.0),^14^ the components of the 2×2 contingency table ‒ True Positives (TP), True Negatives (TN), False Positives (FP), and False Negatives (FN) were calculated using the sensitivity and specificity values reported for the testing set in the original study. The number of TP and TN cases were calculated as:^1^ TP = (Total transfused patients) × Sensitivity;^2^ TN = (Total non-transfused patients) × Specificity. Subsequently, the number of False Positives (FP) and False Negatives (FN) were derived:^3^ FP = (Total non-transfused patients) − TN;^4^ FN = (Total transfused patients) − TP (Supplementary Table 2), rounding results to the nearest integer. When the exact number of transfused patients in the testing set was not reported, it was assumed that the transfused-to-non-transfused ratio matched that of the randomly assigned training set. This assumption applied to two studies, Liu et al.^4^ and Sun et al.^6^ As both studies showed high stability and negligible discrepancies in comparative validation and sensitivity analyses, this assumption likely did not bias the pooled estimates.

For studies reporting medians and interquartile ranges, Luo’s method^20^ was applied to approximate the mean, and Wan’s method^21^ to estimate the SD. Statistical analysis was performed in R (version 4.4.1)^22^ utilizing the Mada^23^ and meta packages.^24^ In accordance with the Cochrane Handbook,^14^ a hierarchical approach was employed to synthesize model performance while accounting for the inherent correlation between sensitivity and specificity.

A bivariate random-effects meta-analysis was implemented using the Reitsma function in the Mada R package, using restricted maximum likelihood estimation. As described by Reitsma et al.,^25^ this model analyzes logit-transformed sensitivity and false positive rates. This hierarchical framework accounts for two levels of variability: within-study sampling error and between-study heterogeneity. This bivariate approach is mathematically equivalent to the Hierarchical Summary Receiver Operating Characteristic (HSROC) model proposed by Rutter and Gatsonis^26^ in the absence of covariates,^27^ which is the case of this meta-analysis. The threshold effect was evaluated primarily through bivariate modeling, as recommended by Reitsma et al.^25^

Heterogeneity was quantified using the Zhou and Dendukuri approach^28^ for bivariate *I²*, which represents the proportion of total variation attributable to between-study inconsistency. We also examined the between-study variance components (τ) for both sensitivity and the false positive rate. Diagnostic performance was visualized using SROC curves. A prediction region could not be traced due to the low number of included studies.^14^^,^^15^ The Area Under the Curve (AUC) was derived directly from the parameters of the bivariate model as a global measure of accuracy. Small-study effects and publication bias were assessed using Deeks’ asymmetry test on the diagnostic odds ratio.^14^

To assess potential bias from rounding methods and stratification assumptions, a comparative analysis was conducted (Supplementary Material). Diagnostic parameters were recalculated from reconstructed 2×2 tables using three rounding strategies (floor, ceiling, and nearest integer) and compared with the metrics originally reported by the authors. Although discrepancies remained negligible across all rounding approaches, rounding to the nearest integer consistently provided the closest approximation to the reported values.

Across four out of five included studies, these discrepancies are negligible. However, the study by Zhou et al.^1^ exhibited substantial discrepancies despite the availability of explicit transfused and non-transfused patient counts for the testing set. Therefore, a supplementary sensitivity analysis was conducted by excluding this study, which did not significantly change results (Supplementary Fig. 1).

## Results

### Study selection

The systematic literature search yielded 549 records (Fig. 1). After the removal of 47 duplicates, 502 unique articles were screened, which led to the exclusion of 479 studies. The full texts of the remaining 23 articles were assessed for eligibility. Of these, 18 studies were excluded for reasons including the use of incorrect index tests (n = 5), publication as congress abstracts or errata (n = 3) or reporting of irrelevant outcomes (n = 10). Ultimately, five studies were included in the meta-analysis.^1^, ^2^, ^3^, ^4^^,^^6^

### Baseline characteristics

This analysis is based on five retrospective studies, encompassing a total cohort of 3,063 patients who underwent various cardiac surgical procedures, including aortic, tricuspid, and mitral valve replacement or implantation, as well as Minimally Invasive Direct Coronary Artery Bypass (MIDCAB).

The pooled weighted mean age of the patient cohort was 59.15 ± 12.67 years, with a sex distribution of 1,879 (61.35%) male and 1,184 (38.65%) female patients. A total of 926 patients (30.23%) received RBC transfusion during the intraoperative period; the proportion of transfused patients ranged from 13.97% to 57.37% among studies. Table 1 presents which AI models were evaluated in each included study and detailed baseline characteristics of patients.

### Risk of bias and certainty of evidence

The methodological quality was consistent among the studies, as detailed in the PROBAST-AI^18^ assessment (Supplementary Fig. 2). The risk of bias was low for the participants in all studies except for Chen et. al.^3^ Predictors and outcome domains were rated as low risk of bias across every study. The analysis domain presented a high risk of bias for all studies, due to lack of model calibration and clinical utility assessment.

According to the GRADE analysis^19^ (Supplementary Fig. 3), the overall certainty of evidence for the test's diagnostic accuracy was rated as low. This determination resulted from downgrading the evidence due to two critical limitations. A serious risk of bias was identified, stemming from the subjective nature of the reference standard. Additionally, there were serious concerns with inconsistency, driven by significant performance heterogeneity among the AI algorithms evaluated.

### Best-performing model included studies and key predictive features

To determine the key predictors for intraoperative RBC transfusion, four of the five studies^1^^,^^3^^,^^4^^,^^6^ employed SHAP (SHapley Additive exPlanations) analysis^29^ on their best-performing model. The remaining study by Cunha et al.^2^ utilized a permutation importance analysis for the same purpose. This technique evaluates feature importance by randomly permuting its values; the resulting change in model performance reflects the degree of reliance on that feature.^30^

The resulting top-performing models and their complete sets of identified predictors varied across the analyses. The study by Sun et al.^6^ identified Extreme Gradient Boosting as the most accurate model, listing hemoglobin, prothrombin time, Body Mass Index (BMI), coronary heart disease with additional comorbidities, and a history of percutaneous coronary intervention as its key predictors. In contrast, Chen et al.^3^ found a Logistic Regression algorithm to be superior, with its most essential predictors being sex, hemoglobin, hematocrit, weight, and age. Categorical Boosting was the optimal model in two different studies, although each highlighted distinct feature sets. The model by Liu et al.^4^ prioritized hematocrit, age, the surgeon responsible for the procedure, weight, and BMI. Meanwhile, the analysis by Zhou et al.^1^ identified sex, hemoglobin, ferritin, Alanine Transaminase (ALT), and total bilirubin as significant factors. Lastly, the statistical approach by Cunha et al.^2^ was limited to five predictors, which were, in order of importance: hemoglobin, age, body surface area, cardiopulmonary bypass, reoperation status, and sex, reporting Logistic Regression as its best-performing AI model. Despite the varied models and predictors, preoperative hemoglobin and patient age consistently emerged as highly significant across multiple studies (Table 2).

**Table 2:** The most important predictors for the best-performing model in each study.

Study	Best performing model	Type of analysis	Predictors (in order of importance)
**Sun, 2024**^6^	Extreme Gradient Boosting	SHAP	Hb, PT, BMI, coronary heart disease, history of PCI, weight, heart rate, PLT, fibrinogen, creatinine, TG, CRP, NT-proBNP, multiple CABG, APTT, apo-B, BV, RBC, Tn, LDL-C.
**Liu, 2021**^4^	Categorical Boosting	SHAP	HCT, age, name of surgeon, weight, BMI, height, anemia, operation type, Hb, RBC, tricuspid valve procedure, PLT, sex, ASA, ALT, AST, mitral valve procedure, PT, INR, creatinine.
**Chen, 2024**^3^	Logistic Regression	SHAP	Sex, Hb, HCT, weight, age, MCH, PT, fibrinogen, stature, operation type, GLB, URIC, TBIL, DBIL, HBDH, RDW-SD, TG, A/G, NYHA, IBIL.
**Cunha, 2023**^2^	Logistic Regression	PI	Hb, age, BSA, CPB, redo, sex.
**Zhou, 2024**^1^	Categorical Boosting	SHAP	Sex, Hb, ALT, ferritin, fibrinogen, TBIL, LVEF, albumin, creatinine, APTT, TAST, PLT, folic acid, vitamin B12, TT, HbA1c.

A/G, albumin/globulin; ALT, alanine transaminase; APTT, activated partial thromboplastin time; ASA, American Society of Anesthesiologists; AST, aspartate transaminase; BMI, body mass index; BSA, body surface area; BV, blood volume; CABG, coronary artery bypass grafting; CPB, cardiopulmonary bypass; CRP, C-reactive protein; DBIL, direct bilirubin; GLB, globulin; Hb, hemoglobin; HbA1c, glycated hemoglobin; HBDH, hydroxybutyrate dehydrogenase; HCT, hematocrit; IBIL, indirect bilirubin; INR, international normalized ratio; LDL-C, low-density lipoprotein cholesterol; LVEF, left ventricular ejection fraction; MCH, mean corpuscular hemoglobin; NT-proBNP, N-terminal pro-brain natriuretic peptide; NYHA, New York Heart Association; PCI, percutaneous coronary intervention; PLT, platelet; PI, permutation importance; PT, prothrombin time; RBC, red blood cell; RDW-SD, red cell distribution width-standard deviation; SHAP, Shapley Additive Explanations; TAST, transferrin saturation; TBIL, total bilirubin; TG, triglycerides; Tn, troponin; TT, thrombin time; URIC, uric acid.

### Performance of AI models

#### Logistic Regression model

**Figure 2:**
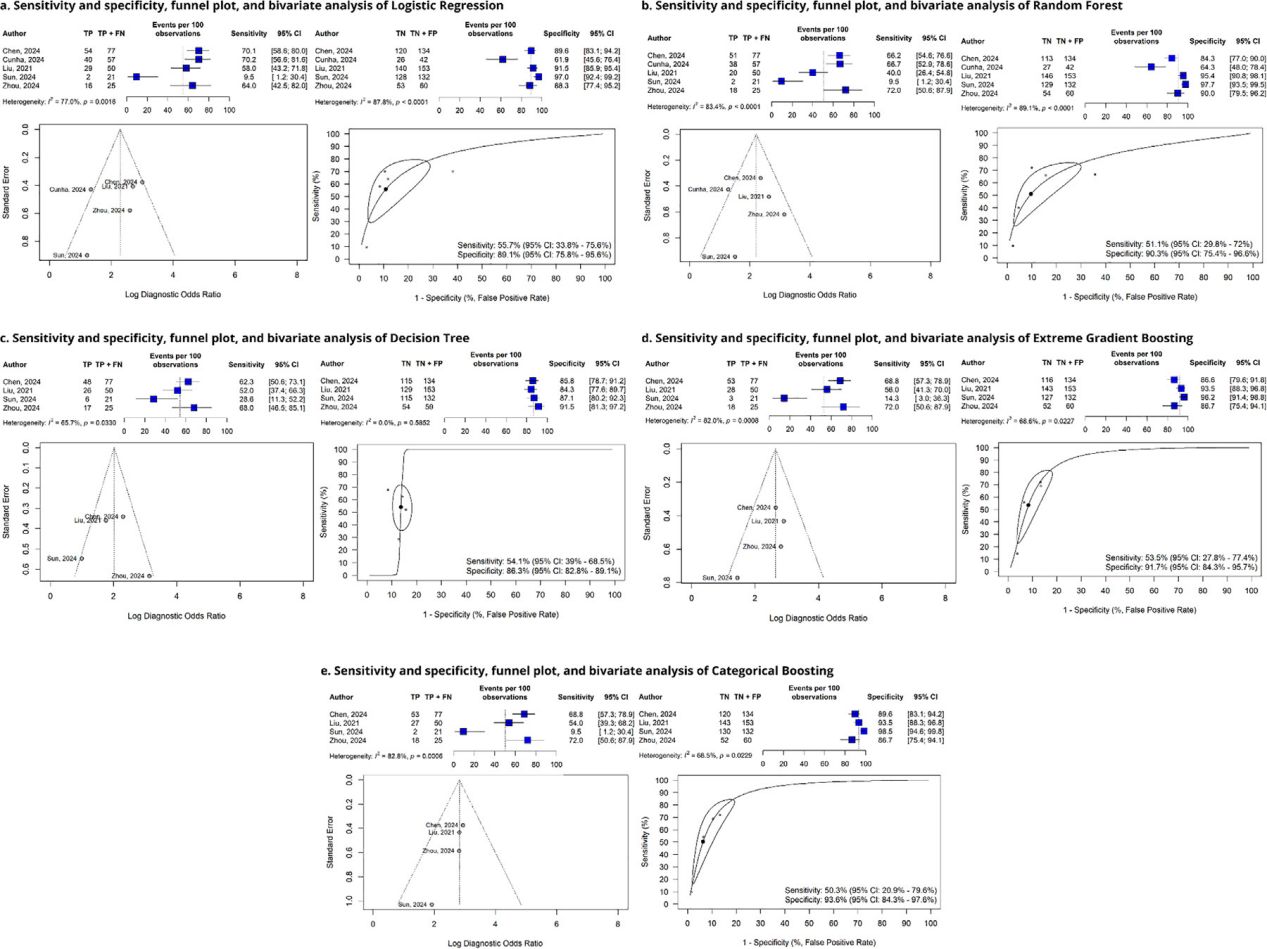
Sensitivity, specificity, funnel plot and bivariate analysis of Logistic Regression (a); Random Forest (b); Decision Tree (c); Gradient Boosting (d); Categorical Boosting (e).

The meta-analysis showed that the Logistic Regression model (Fig. 2a) achieved a pooled sensitivity of 0.557 (95% CI 0.338‒0.756) and a pooled specificity of 0.891 (95% CI 0.758‒0.956). The overall diagnostic accuracy was reflected by an AUC of 0.820 (95% CI 0.635‒0.905). Threshold-related variation was handled through the bivariate random-effects framework, which preserves the two-dimensional nature of the data by jointly analyzing sensitivity and specificity. Within this joint distribution, the model estimated between-study standard deviations for sensitivity (0.954) and specificity (1.037). In the ROC space, the 95% confidence ellipse illustrates the region containing likely combinations of the mean sensitivity and specificity values. The orientation and spread of this ellipse, along with the concave shape of the curve, are consistent with the presence of a threshold effect. As an exploratory finding within the bivariate framework, a correlation of 0.833 was noted between the logit-transformed measures. Regarding broader heterogeneity, the Zhou and Dendukuri^28^ approach estimated a bivariate *I^2^* of 41.5%, pointing to a moderate level of heterogeneity. Deeks’ funnel plot asymmetry test showed no significant asymmetry (*t* = -1.36, p = 0.268).

#### Random Forest

The Random Forest model (Fig. 2b) showed a summary sensitivity of 0.511 (95% CI 0.298‒0.720) and specificity of 0.903 (95% CI 0.754‒0.966), with an AUC of 0.793 (95% CI 0.634‒0.899). Within the bivariate joint distribution, the model estimated between-study standard deviations of 0.954 for sensitivity and 1.208 for specificity. In the ROC space, the orientation and spread of the 95% confidence ellipse are consistent with the presence of a threshold effect. As an exploratory finding, a correlation of 0.928 was estimated between the logit-transformed measures. Bivariate *I²* was 32.7%, reflecting moderate heterogeneity. Deeks’ funnel plot asymmetry test indicated no significant publication bias (*t* = -0.20, p = 0.854).

#### Decision Tree model

For the Decision Tree model (Fig. 2c) the meta-analysis yielded a pooled sensitivity of 0.541 (95% CI 0.390‒0.685) and a pooled specificity of 0.863 (95% CI 0.828‒0.891). The overall diagnostic performance was strong, with an AUC of 0.862 (95% CI 0.595‒0.887). The model estimated between-study standard deviations of 0.515 for sensitivity and 0.016 for specificity. In the ROC space, the orientation and spread of the 95% confidence ellipse are not consistent with the presence of a threshold effect. As an exploratory finding, a correlation of -1.000 was estimated between the logit-transformed measures. Between-study heterogeneity was minimal, with the Zhou and Dendukuri approach estimating a bivariate *I²* of 0%. Evaluation of small-study effects using Deeks’ funnel plot asymmetry test was non-significant (*t* = 0.03, p = 0.980).

#### Extreme Gradient Boosting model

The Extreme Gradient Boosting model (Fig. 2d) showed a pooled sensitivity of 0.535 (95% CI 0.278‒0.774) and specificity of 0.917 (95% CI 0.843‒0.957), with an AUC of 0.886 (95% CI 0.716‒0.929). The model estimated between-study standard deviations of 1.044 for sensitivity and 0.646 for specificity. In the visual ROC space, the orientation and spread of the 95% confidence ellipse are consistent with the presence of a threshold effect. As an exploratory finding, a correlation of 1.000 was estimated between the logit-transformed measures. Bivariate *I²* was 0%, suggesting minimal residual heterogeneity attributable to sampling error and consistent discriminative performance across studies. Deeks’ funnel plot asymmetry test indicated no significant publication bias (*t* = -1.37, p = 0.303).

#### Categorical Boosting model

The Categorical Boosting model showed a pooled sensitivity of 0.503 (95% CI 0.209‒0.796) and specificity of 0.936 (95% CI 0.843‒0.976), with an AUC of 0.892 (95% CI 0.740‒0.943). Within the bivariate joint distribution, the model estimated between-study standard deviations of 1.308 for sensitivity and 0.953 for specificity. In the ROC space, the orientation and spread of the 95% confidence ellipse are consistent with the presence of a threshold effect. As an exploratory finding, a correlation of 1.000 was estimated between the logit-transformed measures. Bivariate *I²* was 0%, suggesting residual heterogeneity is due to sampling error. Deeks’ funnel plot asymmetry test was significant (*t* = -5.33, p = 0.033), indicating potential publication bias or small-study effects affecting pooled estimate reliability.

#### Other models

The diagnostic performance of AI architectures evaluated in fewer than four studies is detailed below, with full outputs available in Supplementary Figures 4‒11. The Adaptive Boosting (n = 3), Gradient Boosting (n = 3), Support Vector Machine (n = 3), and Gradient Boosting (n = 3) demonstrated varied discriminative capabilities. Adaptive Boosting achieved an AUC of 0.867 (95% CI 0.543‒0.897) with a pooled sensitivity of 0.494 (95% CI 0.265‒0.725) and specificity of 0.871 (95% CI 0.833‒0.902); its negligible heterogeneity (*I^2^* = 0%), moderate threshold correlation (*r* = 0.632), and 95% confidence ellipse suggest that variability was driven by random sampling error. Support Vector Machine showed negligible heterogeneity (*I^2^* = 2.1%) and an AUC of 0.698 (95% CI 0.658‒0.883). The Gradient Boosting model presented a moderate level of inconsistency (*I^2^* = 50.9%) despite an AUC of 0.815 (95% CI 0.456‒0.924). The Extra Trees model (n = 3) yielded an AUC of 0.770 (95% CI 0.451‒0.939). Other analyzed models were K Nearest Neighbor (n = 2; AUC = 0.786), Light Gradient Boosting (n = 2; AUC = 0.699), Linear Discriminant Analysis (n = 2; AUC = 0.902), and Multilayer Perceptron (n = 2; AUC = 0.776).

## Discussion

This systematic review and meta-analysis suggests that AI models may have potential for predicting intraoperative RBC transfusion in cardiac surgery; however, this interpretation is limited by low certainty of evidence, substantial heterogeneity, and restricted generalizability. Across five studies with 3,063 patients, models consistently exhibited high specificity (ranging from 86.2% for Decision Tree to 93.6% for Categorical Boosting) and moderate sensitivity (ranging from 50.3% for Categorical Boosting to 55.7% for Logistic Regression). The consistently high specificity observed across models suggests that positive predictions may help identify patients at higher likelihood of receiving transfusion within the studied contexts. However, the moderate-to-low sensitivity indicates that a negative prediction is not enough to rule out the need for blood.^31^ Importantly, the predicted outcome reflects transfusion events determined by local clinical practice rather than a standardized or objective measure of physiological need, which limits clinical interpretability.

### Predictive variables

Predictive features for intraoperative RBC transfusion were heterogeneous, but preoperative hemoglobin and patient age consistently emerged as key risk factors. Other important predictors include sex, hemoglobin, hematocrit, weight, and mean corpuscular hemoglobin, which reflect blood volume; fibrinogen, which reflects coagulation status; and type of surgery, which influences bleeding. The higher prevalence of iron deficiency anemia among females further highlights sex as a relevant risk factor.^32^

### Comparison with established scores

Compared with intraoperative RBC transfusion risk scores such as TRACK and TRUST, which report AUCs of 0.76‒0.80,^12^^,^^33^ the discriminative performance of AI models observed in this review appears numerically higher. However, despite the high specificities and AUCs of AI intraoperative RBC transfusion prediction models, some studies report that in less complex surgeries, such as minimally invasive direct coronary artery bypass, reported AUCs were lower (∼0.73).^1^ This may suggest that model accuracy depends on surgical complexity, institutional practices, and data quality.

Although AI models show numerically higher discrimination than conventional scores, the clinical significance of this difference remains uncertain. In surgical practice, enhanced discrimination is meaningful only if it improves bedside decision-making, which has not yet been demonstrated. AUC differences may reflect the ability of AI models to capture non-linear interactions between risk factors, such as age and preoperative hemoglobin. However, this has not translated into a meaningful change in the sensitivity-specificity trade-off, as sensitivity remains comparable to conventional scores.

### Heterogeneity

Heterogeneity varied across AI models, reflecting differences in study populations, model design, surgical complexity, and local RBC transfusion practices. Threshold effects in some models suggest that performance variability was partly driven by non-standardized transfusion criteria and institutional decision-making. A concave shape of the SROC curve, a diagonally distributed and an elongated spread of the 95% confidence ellipse are indicative of the presence of threshold effect. In contrast, the Decision Tree model showed low heterogeneity and a sharper SROC curve shape, indicating that classical threshold effects along a common ROC curve were not the main source of between-study variability. This apparent consistency may reflect more stable, rule-based decision structures but should be interpreted cautiously, as low heterogeneity could also result from limited dataset variability. Overall, heterogeneity in AI-based intraoperative RBC transfusion prediction appears influenced by both algorithmic characteristics and clinical context, highlighting the need for standardized transfusion criteria and external validation.

### Implications for PBM and resources

These findings suggest a potential exploratory role for AI in perioperative risk stratification. High specificity supports use as a “rule-in” tool for identifying patients at high transfusion risk with a positive result, while low sensitivity limits reliable exclusion, leaving many cases undetected. However, current evidence is insufficient to support these AI models as practice-changing tools in cardiac surgery.

In recent years, increasing awareness of transfusion-associated risks has prompted a paradigm shift towards PBM and more restrictive transfusion strategies.^6^^,^^8^^,^^34^, ^35^, ^36^ However, a gap remains between guidelines and clinical practice. Clinicians often rely on their individual experience to assess intraoperative RBC transfusion needs. Therefore, robust multicenter studies with standardized RBC transfusion criteria and external validation are needed before attempting clinical implementation of AI. Until then, AI models should be regarded as investigational and are not yet suitable for use as clinical decision-support tools.

### Limitations and future prospects

Interpretation of these findings is limited by the available evidence. Given the novelty of this field, few studies met the inclusion criteria, and substantial heterogeneity persisted despite similar predictive features. A major limitation is the use of a subjective reference standard, as the absence of standardized protocols for RBC administration means model outcomes reflect clinician discretion and institutional practices rather than universal physiological thresholds, introducing potential incorporation bias. Furthermore, a relevant methodological limitation involves the reconstruction of 2×2 contingency tables for studies where the exact number of transfused patients in the testing set was not reported. In these instances, it was necessary to assume that the transfused-to-non-transfused ratio matched that of the training set. While comparative validation and sensitivity analyses demonstrated minimal discrepancies, this assumption remains a potential source of imprecision in the pooled estimates that must be explicitly highlighted. The low quality of evidence in GRADE assessment, which primarily derives from the high risk of bias and heterogeneity among studies, also presents a significant limitation.

Future research should prioritize large-scale, standardized, multicenter studies to validate these models in real-world settings, assess their impact on clinical outcomes, intraoperative RBC transfusion rates, and healthcare costs, and ensure generalizability through external validation. Adherence to standardized reporting frameworks, such as TRIPOD,^37^ is essential to enable meaningful comparisons across studies.^2^^,^^3^^,^^6^^,^^9^

## Conclusion

This systematic review and meta-analysis suggest a potential exploratory role for AI in PBM strategies; however, current evidence is insufficient to support their use as a clinical decision-support tool. This interpretation is limited by the low certainty of evidence, heterogeneity across studies, and the use of non-standardized transfusion outcomes. Further large, standardized, and externally validated studies are required before clinical implementation can be considered.

## Data availability statement

This meta-analysis is based on aggregate data extracted from publicly available studies. Requests for additional information should be directed to the corresponding authors of the original publications.

## Study registry (PROSPERO)

CRD420251146515 (https://www.crd.york.ac.uk/PROSPERO/view/CRD420251146515, published September 16, 2025).

## Declaration of generative AI and AI-assisted technologies in the writing process

During the revision of this study, the authors used Gemini 3 in order to reduce the word count and improve readability. The authors reviewed and edited the content as needed, and take full responsibility for the contents of the publication.

## Authors’ contributions

Filipe Giordano Valério: Conceptualization; methodology; formal analysis; investigation; writing-original draft; writing-review & editing. Amanda Carneiro Rodrigues: Data curation; writing-original draft; writing-review & editing. Davi Ricardo Soares Gama de Amorim: Writing-original draft; writing-review & editing; investigation. Roberta Esterque Cantarino: Writing-original draft; writing-review & editing. Glauco Martins de Araujo: Data curation; writing-review & editing. Jimmy Yusuf: Investigation, Data curation; writing-review & editing. Lecticia Vianna Leal Soares Bessa: Investigation; writing-original draft; writing-review & editing. Millena Mendonça Andrade Paes Leme: Writing-original draft; writing-review & editing. Marcella Freire de Campos Euzebio: Writing-original draft; writing-review & editing. Flávio Luiz Seixas: Formal analysis; supervision; writing-review & editing. Alexandra Rezende Assad: Supervision; writing-review & editing. Luis Antonio dos Santos Diego: Supervision; writing-review & editing.

## Conflicts of interest

The authors declare no conflicts of interest.

## Appendix Supplementary materials

Image, application 1

## Footnotes

Supplementary material associated with this article can be found in the online version at doi:10.1016/j.bjane.2026.844782.
