# Supplementary material for: Artificial intelligence in the prediction of intraoperative red blood cell transfusion in cardiac surgery: a systematic review and diagnostic test accuracy meta-analysis
Source: Braz J Anesthesiol. 2026 Jun 25;76(4):844782. doi: 10.1016/j.bjane.2026.844782 (PMC13379987; doi:10.1016/j.bjane.2026.844782)

**BJAN-D-25-00666_Supplementary Material**

**Supplementary Table 1** Search strategies for each database.

| **PubMed (182 results from inception to April 15th, 2025)** | ("Artificial Intelligence" OR "artificial intelligence"[mh] OR "AI" OR "Machine learning") AND ("Perioperative Transfusion" OR "Perioperative Transfusions" OR "Intraoperative Transfusion" OR "Intraoperative Transfusions" OR "Postoperative Transfusion" OR "Postoperative Transfusions" OR "Blood Transfusion" OR "blood transfusion"[mh] OR "Blood Transfusions" OR "Red Blood Cell Transfusion" OR "Red Blood Cell Transfusions" OR "Erythrocyte Transfusion" OR "Erythrocyte Transfusions" OR "Blood Component Transfusion" OR "Blood Component Transfusions" OR Transfusion) AND ("Cardiac Surg*" OR "Cardiovascular Surg*" OR "Coronary Artery Bypass Grafting" OR "Heart Surgery" OR "Valve Replacement" OR "Cardiac Operations" OR "Aortic Surgery" OR "Heart Transplantation" OR "Cardiac Valve Surgery" OR "Congenital Heart Surgery" OR "Coronary Artery Disease Surgery" OR "Aortic Valve Replacement" OR "Mitral Valve Surgery" OR "Endocarditis Surgery" OR "Coronary Artery Bypass Surgery" OR "CABG" OR "Percutaneous Coronary Intervention" OR "Stent Insertion" OR "Percutaneous Coronary Angioplasty" OR "Valve Repair" OR "Left Ventricular Assist Device" OR "Heart Valve Surgery" OR "Aortic Dissection Surgery" OR "Tricuspid Valve Surgery" OR "Coronary Artery Disease Surgery" OR "Ventricular Septal Defect Surgery" OR "Tetralogy of Fallot Surgery" OR "Patent Ductus Arteriosus Surgery" OR "Atrial Septal Defect Surgery" OR "Endocardial Pacing" OR "Heart Transplantation Surgery" OR "Pulmonary Valve Surgery" OR "Pulmonary Artery Surgery" OR "Pulmonary Hypertension Surgery" OR "Reoperative Coronary Bypass Surgery" OR "Mitral Valve Prolapse Surgery" OR "Heart Failure Surgery" OR "Cardiac Pacemaker Implantation") |
| --- | --- |
| **Embase (366 results from inception to April 15th, 2025)** | ('artificial intelligence'/exp OR 'artificial intelligence' OR 'ai' OR 'machine learning'/exp OR 'deep learning'/exp OR 'predictive model*') AND ('transfusion'/exp OR 'transfusion' OR 'blood transfusion'/exp OR 'red blood cell transfusion'/exp OR 'red blood cell transfusion' OR 'rbc transfusion' OR 'erythrocyte transfusion'/exp OR 'erythrocyte transfusion' OR 'platelet transfusion'/exp OR 'platelet transfusion' OR 'plasma transfusion'/exp OR 'plasma transfusion' OR 'blood component transfusion'/exp OR 'blood component transfusion' OR 'perioperative transfusion' OR 'intraoperative transfusion' OR 'postoperative transfusion' OR 'massive transfusion'/exp OR 'massive transfusion' OR 'transfusion requirement*') AND ('cardiac surgery'/exp OR 'cardiac surgery' OR 'heart surg*' OR 'cardiac surg*' OR 'thoracic surg*' OR 'open heart surg*' OR 'closed heart surg*' OR 'minimally invasive cardiac surg*' OR 'coronary artery bypass'/exp OR 'coronary artery bypass' OR 'valve surg*' OR 'aortic surg*' OR 'aneurysm repair'/exp OR 'ascending aorta surg*' OR 'aortic root replac*' OR 'ross procedure'/exp OR 'ross procedure' OR 'congenital heart surg*' OR 'septal defect repair' OR 'atrial septal defect repair'/exp OR 'atrial septal defect repair' OR 'ventricular septal defect repair'/exp OR 'ventricular septal defect repair' OR 'tetralogy of fallot repair'/exp OR 'tetralogy of fallot repair' OR 'fontan procedure'/exp OR 'fontan procedure' OR 'norwood procedure'/exp OR 'norwood procedure' OR 'transposition repair' OR 'mitral valve repair'/exp OR 'mitral valve repair' OR 'mitral valve replacement'/exp OR 'mitral valve replacement' OR 'aortic valve repair'/exp OR 'aortic valve repair' OR 'aortic valve replacement'/exp OR 'aortic valve replacement' OR 'tricuspid valve repair'/exp OR 'tricuspid valve repair' OR 'tricuspid valve replacement'/exp OR 'tricuspid valve replacement' OR 'pulmonary valve repair'/exp OR 'pulmonary valve repair' OR 'pulmonary valve replacement'/exp OR 'pulmonary valve replacement' OR 'transcatheter valve'/exp OR 'transcatheter valve' OR 'tavr' OR 'tmvr' OR 'maze procedure'/exp OR 'maze procedure' OR 'myectomy'/exp OR 'myectomy' OR 'heart transplant*' OR 'cardiac assist device'/exp OR 'cardiac assist device' OR 'ventricular assist device'/exp OR 'ventricular assist device' OR 'vad implantation' OR 'lvad'/exp OR 'lvad' OR 'rvad' OR 'ecmo' OR 'extracorporeal membrane oxygenation'/exp OR 'extracorporeal membrane oxygenation' OR 'cardiac reoperation' OR 'redo cardiac surg*') |
| **Cochrane Library (1 result from inception to April 15th, 2025)** | ("Artificial Intelligence" OR “artificial intelligence”[mh] OR "Machine learning" OR "Deep Learning") AND ("Perioperative Transfusion" OR "Perioperative Transfusions" OR "Intraoperative Transfusion" OR "Intraoperative Transfusions" OR "Blood Transfusion" OR “blood transfusion”[mh] OR "Blood Transfusions" OR "Red Blood Cell Transfusion" OR "Red Blood Cell Transfusions" OR "Erythrocyte Transfusion" OR "Erythrocyte Transfusions" OR "Blood Component Transfusion" OR "Blood Component Transfusions") AND ("Cardiac Surgery" OR "Cardiovascular Surgery" OR "Coronary Artery Bypass Grafting" OR "Heart Surgery" OR "Valve Replacement" OR "Cardiac Operations" OR "Aortic Surgery" OR "Heart Transplantation" OR "Cardiac Valve Surgery" OR "Congenital Heart Surgery" OR "Coronary Artery Disease Surgery" OR "Aortic Valve Replacement" OR "Mitral Valve Surgery" OR "Endocarditis Surgery" OR "Coronary Artery Bypass Surgery" OR "CABG" OR "Percutaneous Coronary Intervention" OR "Stent Insertion" OR "Percutaneous Coronary Angioplasty" OR "Valve Repair" OR "Left Ventricular Assist Device" OR "Heart Valve Surgery" OR "Aortic Dissection Surgery" OR "Tricuspid Valve Surgery" OR "Coronary Artery Disease Surgery" OR "Ventricular Septal Defect Surgery" OR "Tetralogy of Fallot Surgery" OR "Patent Ductus Arteriosus Surgery" OR "Atrial Septal Defect Surgery" OR "Endocardial Pacing" OR "Heart Transplantation Surgery" OR "Pulmonary Valve Surgery" OR "Pulmonary Artery Surgery" OR "Atrial Fibrillation Surgery" OR "Cardiac Pacemaker Implantation" OR "Heart Failure Surgery" OR "Mitral Valve Prolapse Surgery" OR "Reoperative Coronary Bypass Surgery" OR "Pulmonary Hypertension Surgery") |

**Supplementary Table 2** 2x2 matrix of each model in the included studies using the nearest integer rounding method. (D+ = transfused patients; D- = non-transfused patients; T+ = predicted positive by AI; T- = predicted negative by AI).

**2a.** Sun et al.

| **Sun, 2024 - 10.1159/000540349** | | | | |
| --- | --- | --- | --- | --- |
| **n(U)** | **n(D+) TEST** | **n(D-) TEST** | **Training set** | **Testing set** |
| **766** | **21** | **132** | **613** | **153** |
| **Extreme Gradient Boosting** | | | | |
|  | **D+** | **D-** |  |  |
| **T+** | **3** | **5** | **8** |  |
| **T-** | **18** | **127** | **145** |  |
|  | **21** | **132** | **153** |  |
| **Gradient Boosting** | | | | |
|  | **D+** | **D-** |  |  |
| **T+** | **4** | **8** | **12** |  |
| **T-** | **17** | **124** | **141** |  |
|  | **21** | **132** | **153** |  |
| **Extra Trees** | | | | |
|  | **D+** | **D-** |  |  |
| **T+** | **1** | **2** | **3** |  |
| **T-** | **20** | **130** | **150** |  |
|  | **21** | **132** | **153** |  |
| **Logistic Regression** | | | | |
|  | **D+** | **D-** |  |  |
| **T+** | **2** | **4** | **6** |  |
| **T-** | **19** | **128** | **147** |  |
|  | **21** | **132** | **153** |  |
| **Categorical Boosting** | | | | |
|  | **D+** | **D-** |  |  |
| **T+** | **2** | **2** | **4** |  |
| **T-** | **19** | **130** | **149** |  |
|  | **21** | **132** | **153** |  |
| **Adaptive Boosting** | | | | |
|  | **D+** | **D-** |  |  |
| **T+** | **5** | **16** | **21** |  |
| **T-** | **16** | **116** | **132** |  |
|  | **21** | **132** | **153** |  |
| **Linear Discriminant Analysis** | | | | |
|  | **D+** | **D-** |  |  |
| **T+** | **4** | **9** | **13** |  |
| **T-** | **17** | **123** | **140** |  |
|  | **21** | **132** | **153** |  |
| **Random Forest** | | | | |
|  | **D+** | **D-** |  |  |
| **T+** | **2** | **3** | **5** |  |
| **T-** | **19** | **129** | **148** |  |
|  | **21** | **132** | **153** |  |
| **Decision Tree** | | | | |
|  | **D+** | **D-** |  |  |
| **T+** | **6** | **17** | **23** |  |
| **T-** | **15** | **115** | **130** |  |
|  | **21** | **132** | **153** |  |
| **K Nearest Neighbor** | | | | |
|  | **D+** | **D-** |  |  |
| **T+** | **2** | **3** | **5** |  |
| **T-** | **19** | **129** | **148** |  |
|  | **21** | **132** | **153** |  |

**2b.** Liu et al.

| **Liu, 2021 - DOI 10.21037/atm-20-7375** | | | | |
| --- | --- | --- | --- | --- |
| **n(U)** | **n(D+) TEST** | **n(D-) TEST** | **Training set** | **Testing set** |
| **677** | **50** | **154** | **473** | **204** |
| **Categorical Boosting** | | | | |
|  | **D+** | **D-** |  |  |
| **T+** | **27** | **10** | **37** |  |
| **T-** | **23** | **144** | **167** |  |
|  | **50** | **154** | **204** |  |
| **Light Gradient Boosting** | | | | |
|  | **D+** | **D-** |  |  |
| **T+** | **29** | **11** | **40** |  |
| **T-** | **21** | **143** | **164** |  |
|  | **50** | **154** | **204** |  |
| **Extreme Gradient Boosting** | | | | |
|  | **D+** | **D-** |  |  |
| **T+** | **28** | **10** | **38** |  |
| **T-** | **22** | **144** | **166** |  |
|  | **50** | **154** | **204** |  |
| **Gradient Boosting** | | | | |
|  | **D+** | **D-** |  |  |
| **T+** | **27** | **11** | **38** |  |
| **T-** | **23** | **143** | **166** |  |
|  | **50** | **154** | **204** |  |
| **Extra Trees** | | | | |
|  | **D+** | **D-** |  |  |
| **T+** | **22** | **9** | **31** |  |
| **T-** | **28** | **145** | **173** |  |
|  | **50** | **154** | **204** |  |
| **Logistic Regression** | | | | |
|  | **D+** | **D-** |  |  |
| **T+** | **29** | **13** | **42** |  |
| **T-** | **21** | **141** | **162** |  |
|  | **50** | **154** | **204** |  |
| **Linear Discriminant Analysis** | | | | |
|  | **D+** | **D-** |  |  |
| **T+** | **29** | **15** | **44** |  |
| **T-** | **21** | **139** | **160** |  |
|  | **50** | **154** | **204** |  |
| **Random Forest** | | | | |
|  | **D+** | **D-** |  |  |
| **T+** | **20** | **7** | **27** |  |
| **T-** | **30** | **147** | **177** |  |
|  | **50** | **154** | **204** |  |
| **Adaptive Boosting** | | | | |
|  | **D+** | **D-** |  |  |
| **T+** | **27** | **18** | **45** |  |
| **T-** | **23** | **136** | **159** |  |
|  | **50** | **154** | **204** |  |
| **Naive Bayes** | | | | |
|  | **D+** | **D-** |  |  |
| **T+** | **41** | **50** | **91** |  |
| **T-** | **9** | **104** | **113** |  |
|  | **50** | **154** | **204** |  |
| **K Nearest Neighbor** | | | | |
|  | **D+** | **D-** |  |  |
| **T+** | **16** | **12** | **28** |  |
| **T-** | **34** | **142** | **176** |  |
|  | **50** | **154** | **204** |  |
| **Decision Tree** | | | | |
|  | **D+** | **D-** |  |  |
| **T+** | **26** | **24** | **50** |  |
| **T-** | **24** | **130** | **154** |  |
|  | **50** | **154** | **204** |  |
| **Quadratic Discriminant Analysis** | | | | |
|  | **D+** | **D-** |  |  |
| **T+** | **44** | **97** | **141** |  |
| **T-** | **6** | **57** | **63** |  |
|  | **50** | **154** | **204** |  |

**2c.** Chen et al.

| **Chen, 2024 - DOI 10.7754/Clin.Lab.2023.230930** | | | | |
| --- | --- | --- | --- | --- |
| **n(U)** | **n(D+) TEST** | **n(D-) TEST** | **Training set** | **Testing set** |
| **702** | **77** | **134** | **491** | **211** |
| **Logistic Regression** | | | | |
|  | **D+** | **D-** |  |  |
| **T+** | **54** | **14** | **174** |  |
| **T-** | **23** | **120** | **37** |  |
|  | **77** | **134** | **211** |  |
| **Categorical Boosting** | | | | |
|  | **D+** | **D-** |  |  |
| **T+** | **53** | **14** | **67** |  |
| **T-** | **24** | **120** | **144** |  |
|  | **77** | **134** | **211** |  |
| **Extra Trees** | | | | |
|  | **D+** | **D-** |  |  |
| **T+** | **55** | **15** | **70** |  |
| **T-** | **22** | **119** | **141** |  |
|  | **77** | **134** | **211** |  |
| **Gaussian Naive Bayes** | | | | |
|  | **D+** | **D-** |  |  |
| **T+** | **58** | **18** | **76** |  |
| **T-** | **19** | **116** | **135** |  |
|  | **77** | **134** | **211** |  |
| **Multilayer Perceptron** | | | | |
|  | **D+** | **D-** |  |  |
| **T+** | **53** | **16** | **69** |  |
| **T-** | **24** | **118** | **142** |  |
|  | **77** | **134** | **211** |  |
| **Adaptive Boosting** | | | | |
|  | **D+** | **D-** |  |  |
| **T+** | **51** | **20** | **71** |  |
| **T-** | **26** | **114** | **140** |  |
|  | **77** | **134** | **211** |  |
| **Random Forest** | | | | |
|  | **D+** | **D-** |  |  |
| **T+** | **51** | **21** | **72** |  |
| **T-** | **26** | **113** | **139** |  |
|  | **77** | **134** | **211** |  |
| **Extreme Gradient Boosting** | | | | |
|  | **D+** | **D-** |  |  |
| **T+** | **53** | **18** | **71** |  |
| **T-** | **24** | **116** | **140** |  |
|  | **77** | **134** | **211** |  |
| **Light Gradient Boosting** | | | | |
|  | **D+** | **D-** |  |  |
| **T+** | **49** | **24** | **73** |  |
| **T-** | **28** | **110** | **138** |  |
|  | **77** | **134** | **211** |  |
| **Bernoulli Naive Bayes** | | | | |
|  | **D+** | **D-** |  |  |
| **T+** | **51** | **18** | **69** |  |
| **T-** | **26** | **116** | **142** |  |
|  | **77** | **134** | **211** |  |
| **Support Vector Machine** | | | | |
|  | **D+** | **D-** |  |  |
| **T+** | **55** | **20** | **75** |  |
| **T-** | **22** | **114** | **136** |  |
|  | **77** | **134** | **211** |  |
| **Gradient Boosting** | | | | |
|  | **D+** | **D-** |  |  |
| **T+** | **45** | **25** | **70** |  |
| **T-** | **32** | **109** | **141** |  |
|  | **77** | **134** | **211** |  |
| **Decision Tree** | | | | |
|  | **D+** | **D-** |  |  |
| **T+** | **48** | **19** | **67** |  |
| **T-** | **29** | **115** | **144** |  |
|  | **77** | **134** | **211** |  |

**2d.** Cunha et al.

| **Cunha, 2024 - DOI 10.21470/1678-9741-2023-0212** | | | | |
| --- | --- | --- | --- | --- |
| **n(U)** | **n(D+) TEST** | **n(D-) TEST** | **Training set** | **Testing set** |
| 495 | 57 | 42 | 396 | 99 |
| **Logistic Regression** | | | | |
|  | **D+** | **D-** |  |  |
| **T+** | 40 | 16 | 56 |  |
| **T-** | 17 | 26 | 43 |  |
|  | 57 | 42 | 99 |  |
| **Multilayer Perceptron** | | | | |
|  | **D+** | **D-** |  |  |
| **T+** | 45 | 20 | 65 |  |
| **T-** | 12 | 22 | 34 |  |
|  | 57 | 42 | 99 |  |
| **Random Forest** | | | | |
|  | **D+** | **D-** |  |  |
| **T+** | 38 | 15 | 53 |  |
| **T-** | 19 | 27 | 46 |  |
|  | 57 | 42 | 99 |  |
| **Support Vector Machine** | | | | |
|  | **D+** | **D-** |  |  |
| **T+** | 40 | 16 | 56 |  |
| **T-** | 17 | 26 | 43 |  |
|  | 57 | 42 | 99 |  |
| **TRACK Score** | | | | |
|  | **D+** | **D-** |  |  |
| **T+** | 34 | 14 | 48 |  |
| **T-** | 23 | 28 | 51 |  |
|  | 57 | 42 | 99 |  |
| **TRUST Score** | | | | |
|  | **D+** | **D-** |  |  |
| **T+** | 43 | 23 | 66 |  |
| **T-** | 14 | 19 | 33 |  |
|  | 57 | 42 | 99 |  |

**2e.** Zhou et al.

| **Zhou, 2024 - DOI 10.3389/fcvm.2024.1344170** | | | | |
| --- | --- | --- | --- | --- |
| **n(U)** | **n(D+) TEST** | **n(D-) TEST** | **Training set** | **Testing set** |
| 423 | 25 | 60 | 338 | 85 |
| **Extreme Gradient Boosting** | | | | |
|  | **D+** | **D-** |  |  |
| **T+** | 18 | 8 | 26 |  |
| **T-** | 7 | 52 | 59 |  |
|  | 25 | 60 | 85 |  |
| **Random Forest** | | | | |
|  | **D+** | **D-** |  |  |
| **T+** | 18 | 6 | 24 |  |
| **T-** | 7 | 54 | 61 |  |
|  | 25 | 60 | 85 |  |
| **Decision Tree** | | | | |
|  | **D+** | **D-** |  |  |
| **T+** | 17 | 6 | 23 |  |
| **T-** | 8 | 54 | 62 |  |
|  | 25 | 60 | 85 |  |
| **Categorical Boosting** | | | | |
|  | **D+** | **D-** |  |  |
| **T+** | 18 | 8 | 26 |  |
| **T-** | 7 | 52 | 59 |  |
|  | 25 | 60 | 85 |  |
| **Support Vector Machine** | | | | |
|  | **D+** | **D-** |  |  |
| **T+** | 17 | 7 | 24 |  |
| **T-** | 8 | 53 | 61 |  |
|  | 25 | 60 | 85 |  |
| **Logistic Regression** | | | | |
|  | **D+** | **D-** |  |  |
| **T+** | 16 | 7 | 23 |  |
| **T-** | 9 | 53 | 62 |  |
|  | 25 | 60 | 85 |  |

**Supplementary Figure 1** Sensitivity analysis for the models reported by Zhou et al.


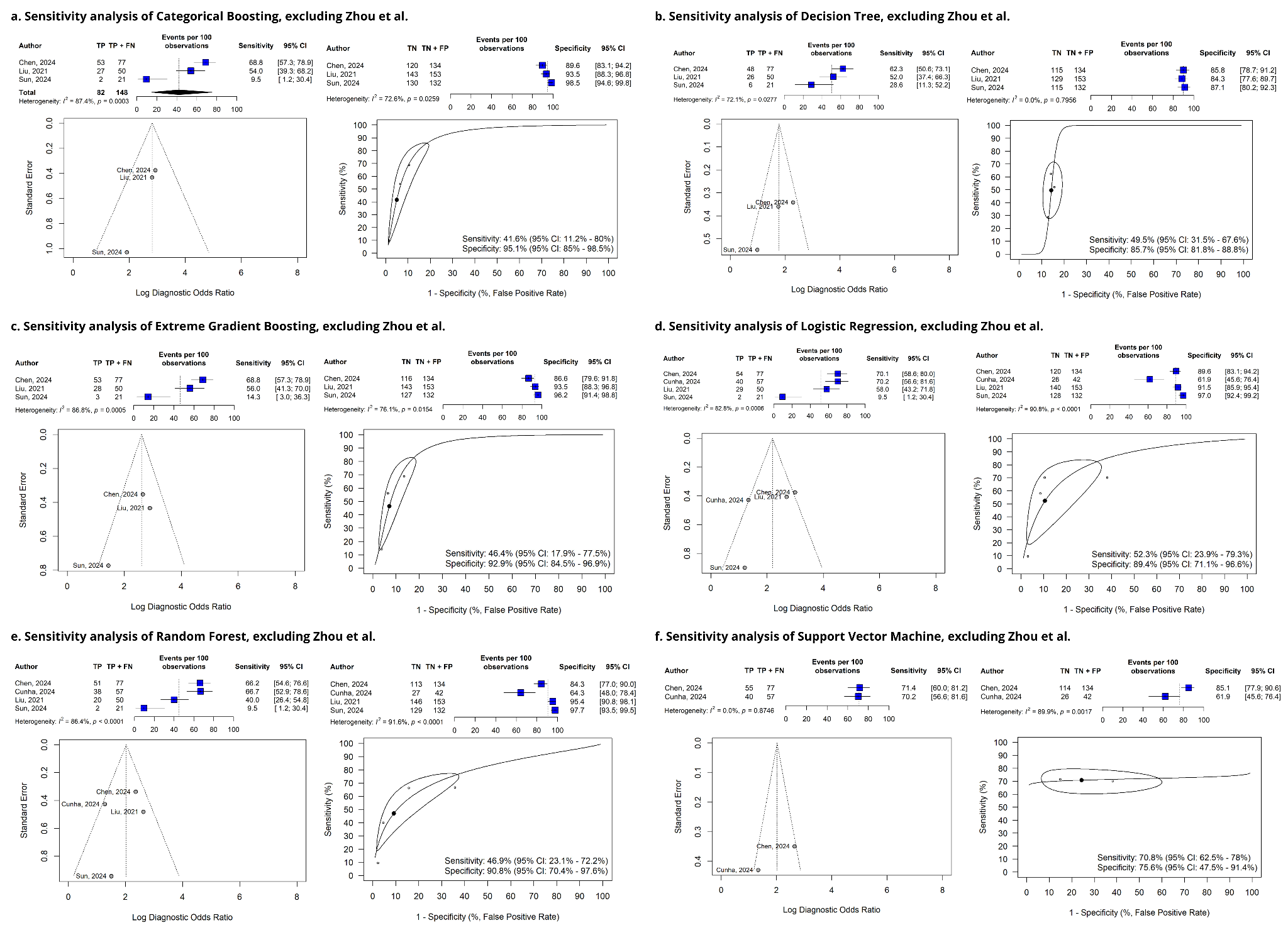


**Supplementary Figure 2** PROBAST-AI traffic light and summary plots of included studies.

**
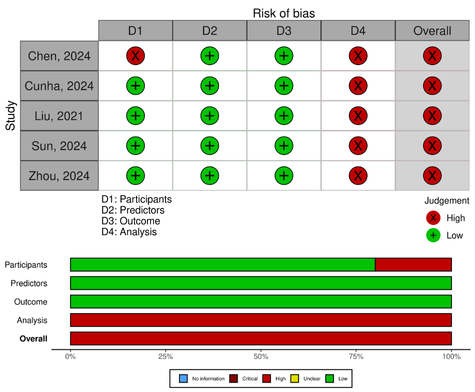
**

**Supplementary Figure 3** GRADE assessment of diagnostic test.


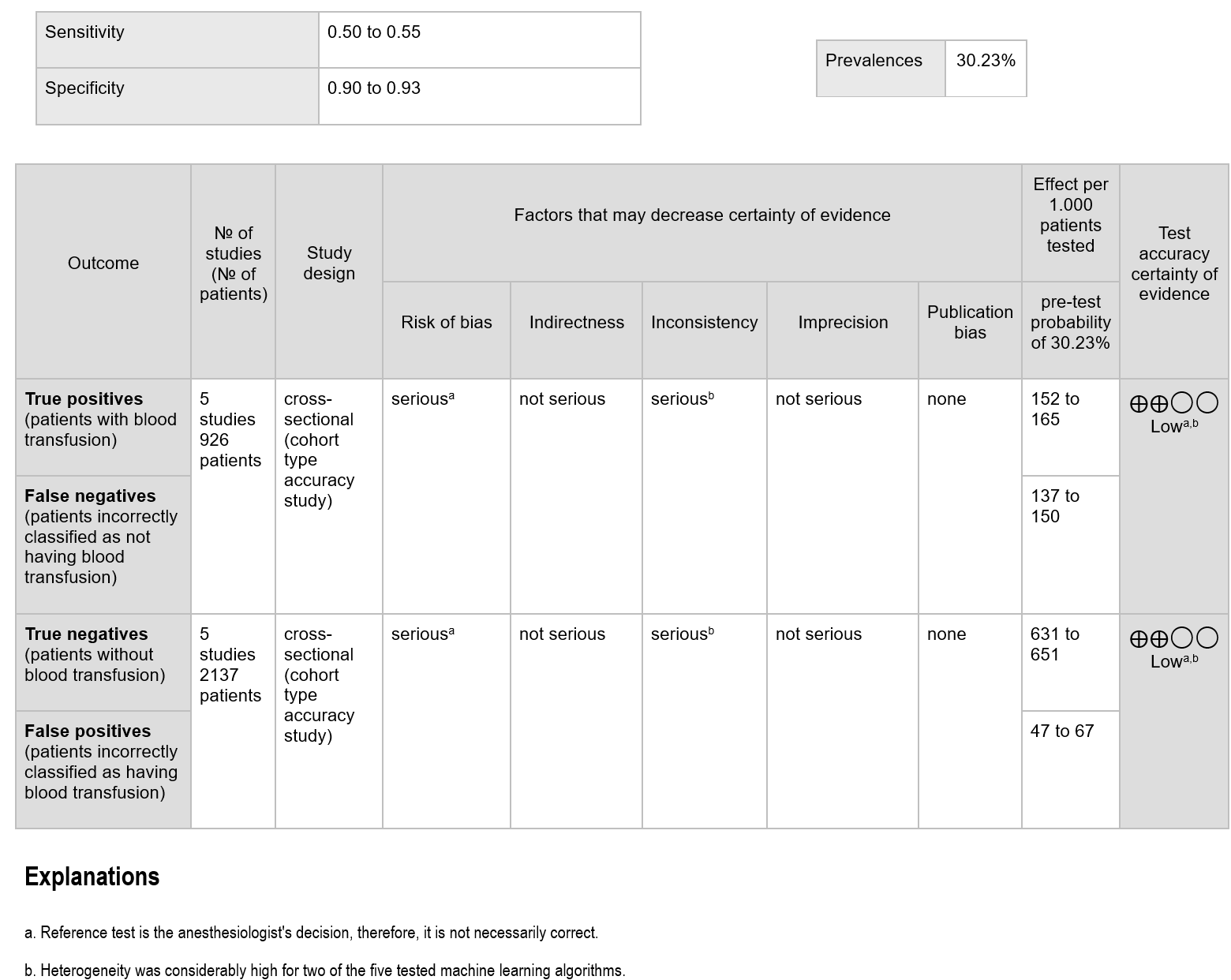


**Supplementary Figure 4** Sensitivity, specificity, funnel plot and bivariate analysis of Adaptive Boosting.
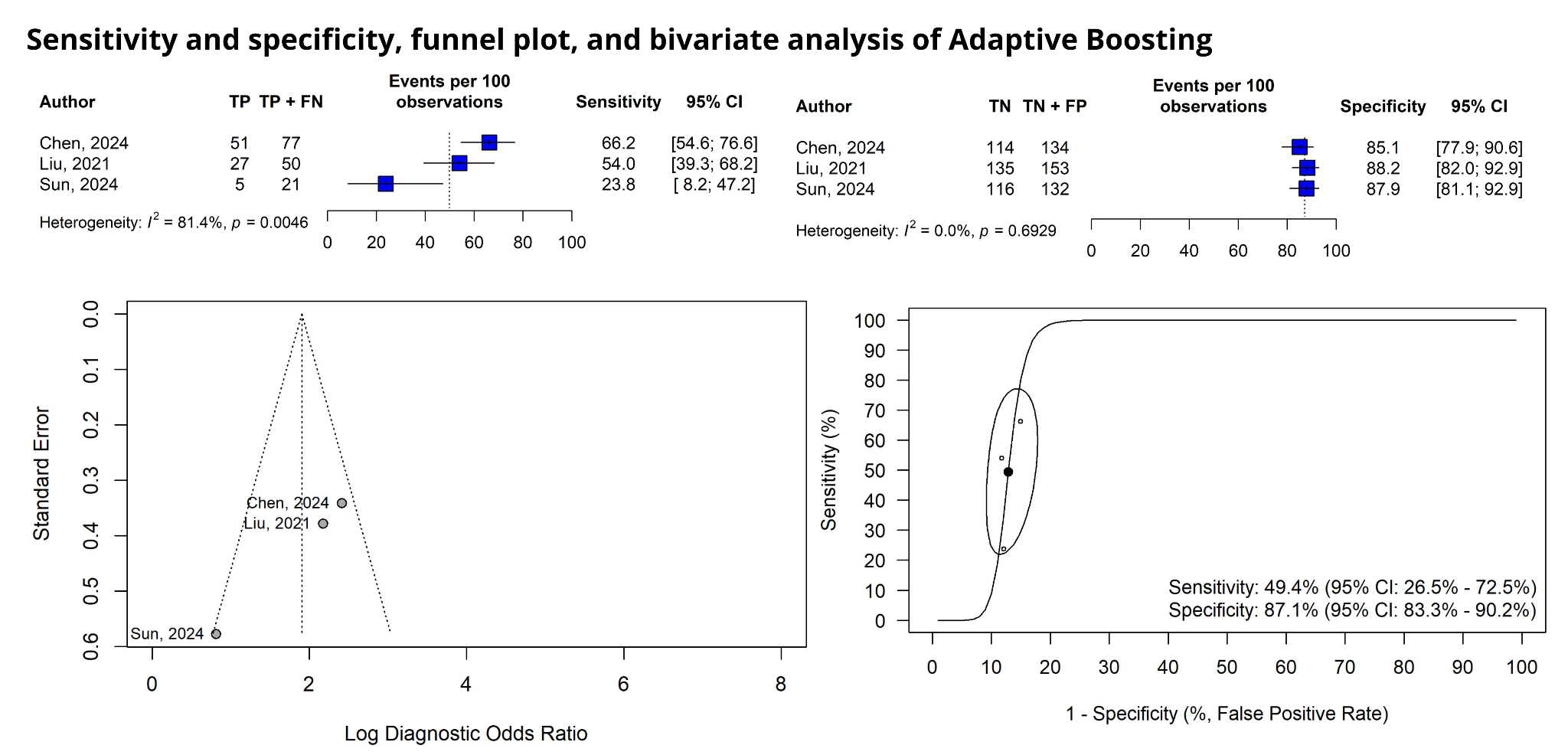


**Supplementary Figure 5** Sensitivity, specificity, funnel plot and bivariate analysis of Extra Trees.


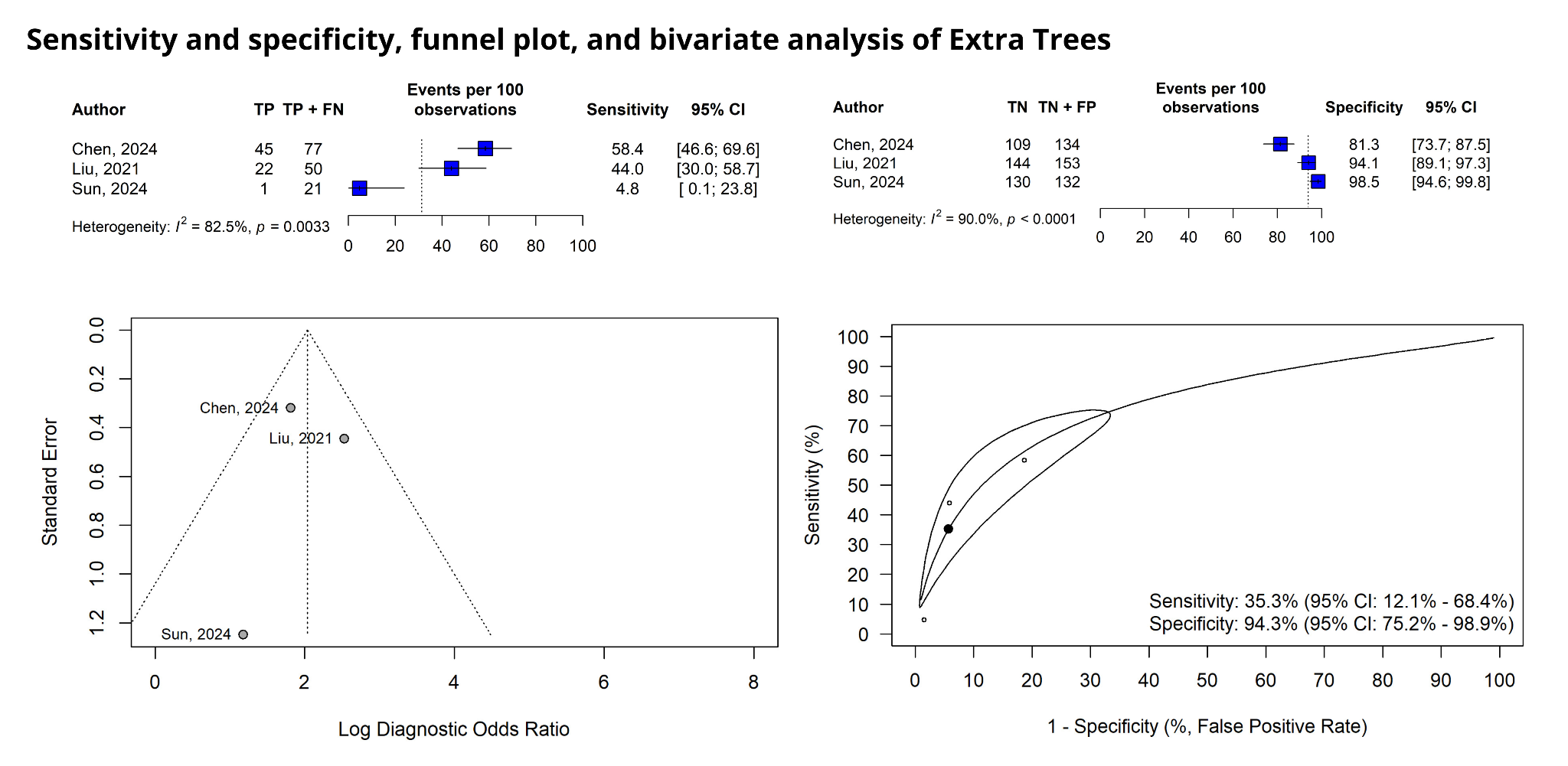


**Supplementary Figure 6** Sensitivity, specificity, funnel plot and bivariate analysis of Multilayer Perceptron.


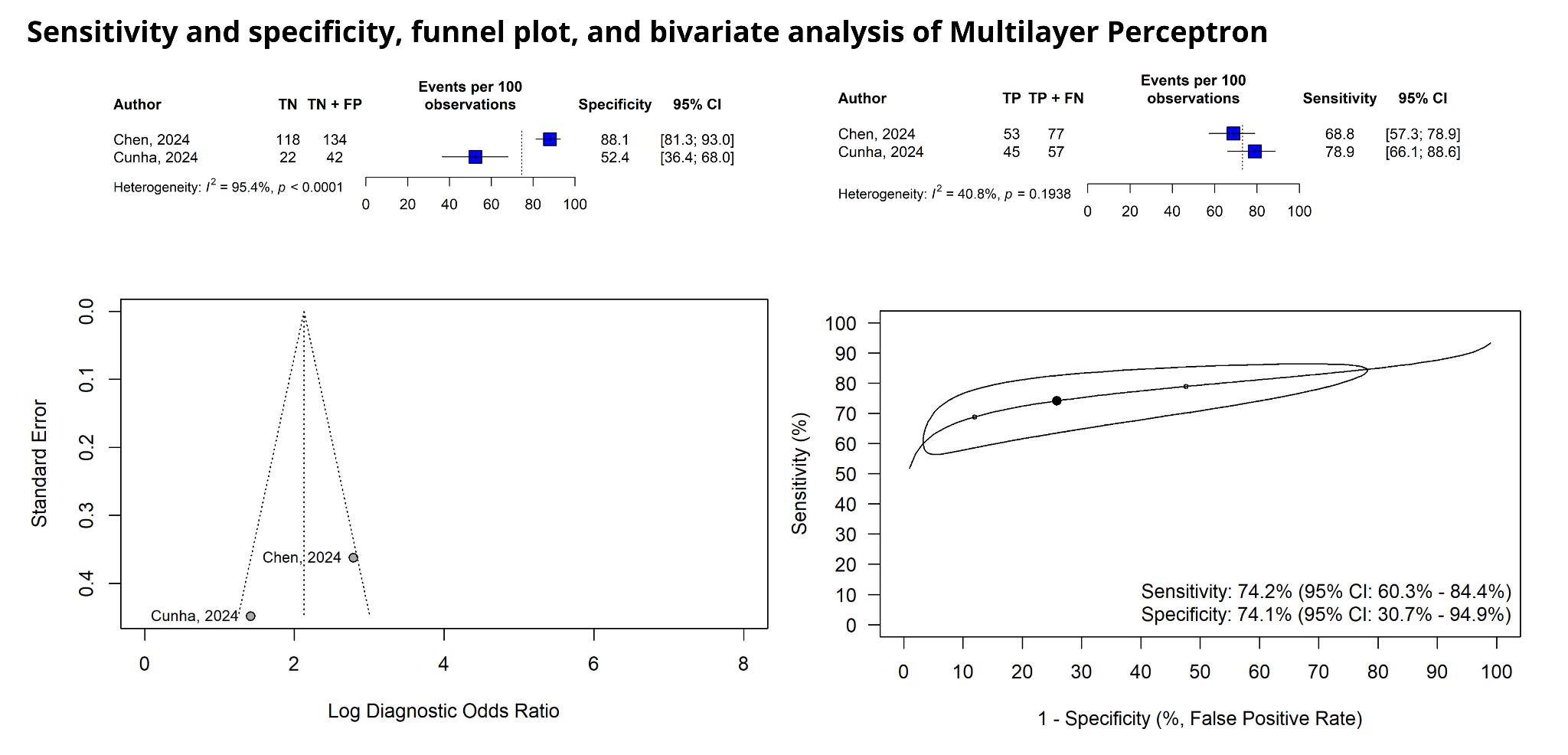


**Supplementary Figure 7** Sensitivity, specificity, funnel plot and bivariate analysis of Gradient Boosting.


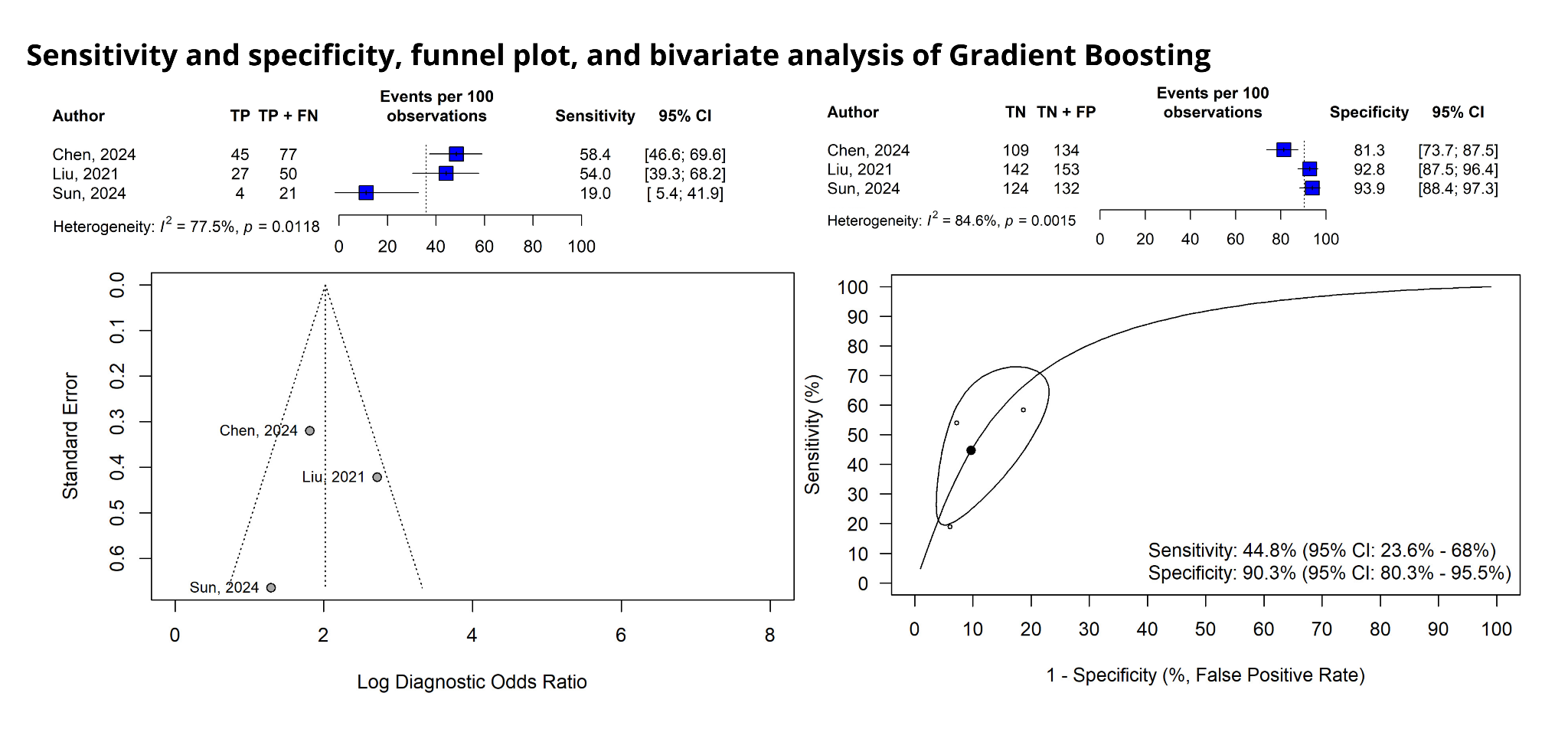


**Supplementary Figure 8** Sensitivity, specificity, funnel plot and bivariate analysis of K Nearest Neighbor.


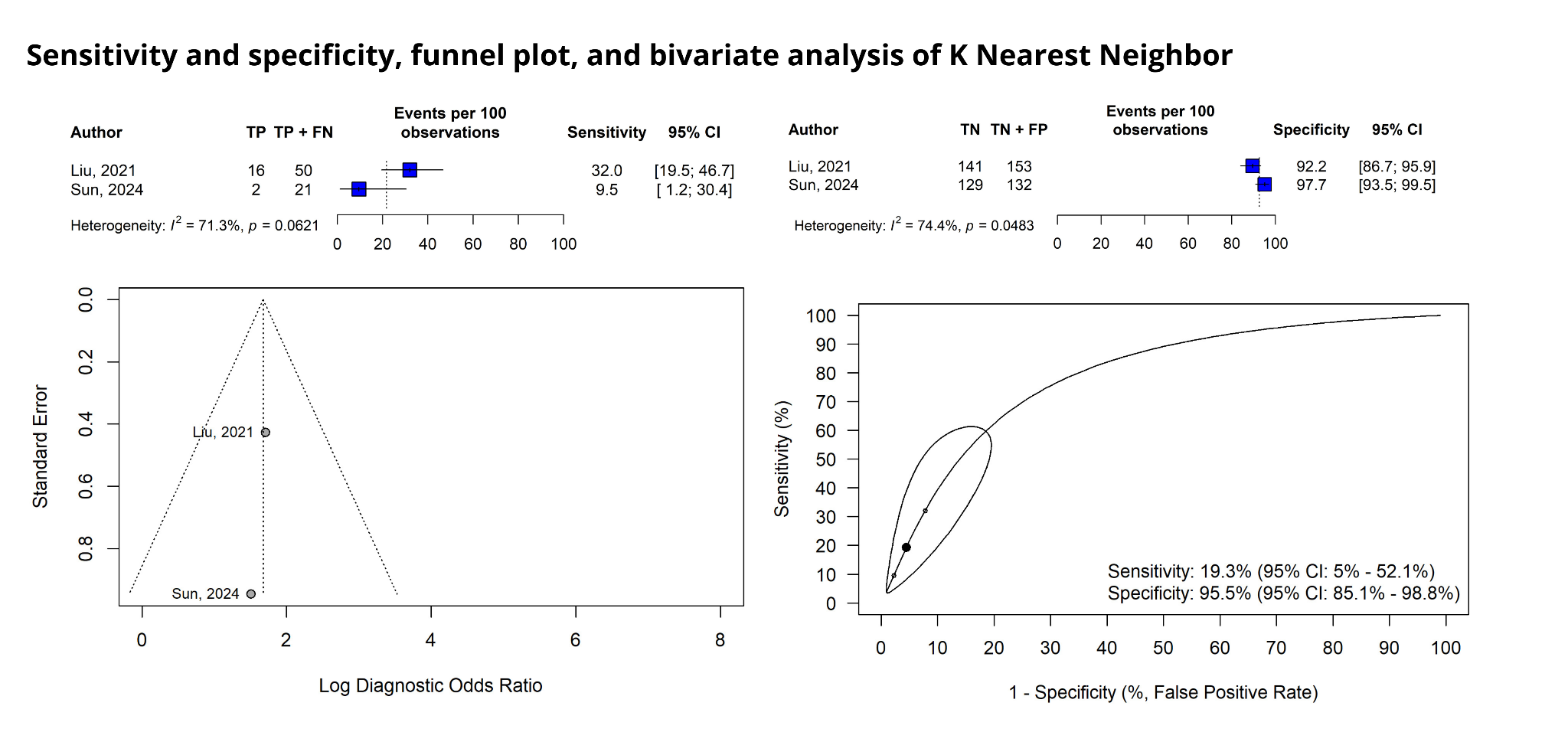


**Supplementary Figure 9** Sensitivity, specificity, funnel plot and bivariate analysis of Support Vector Machine.


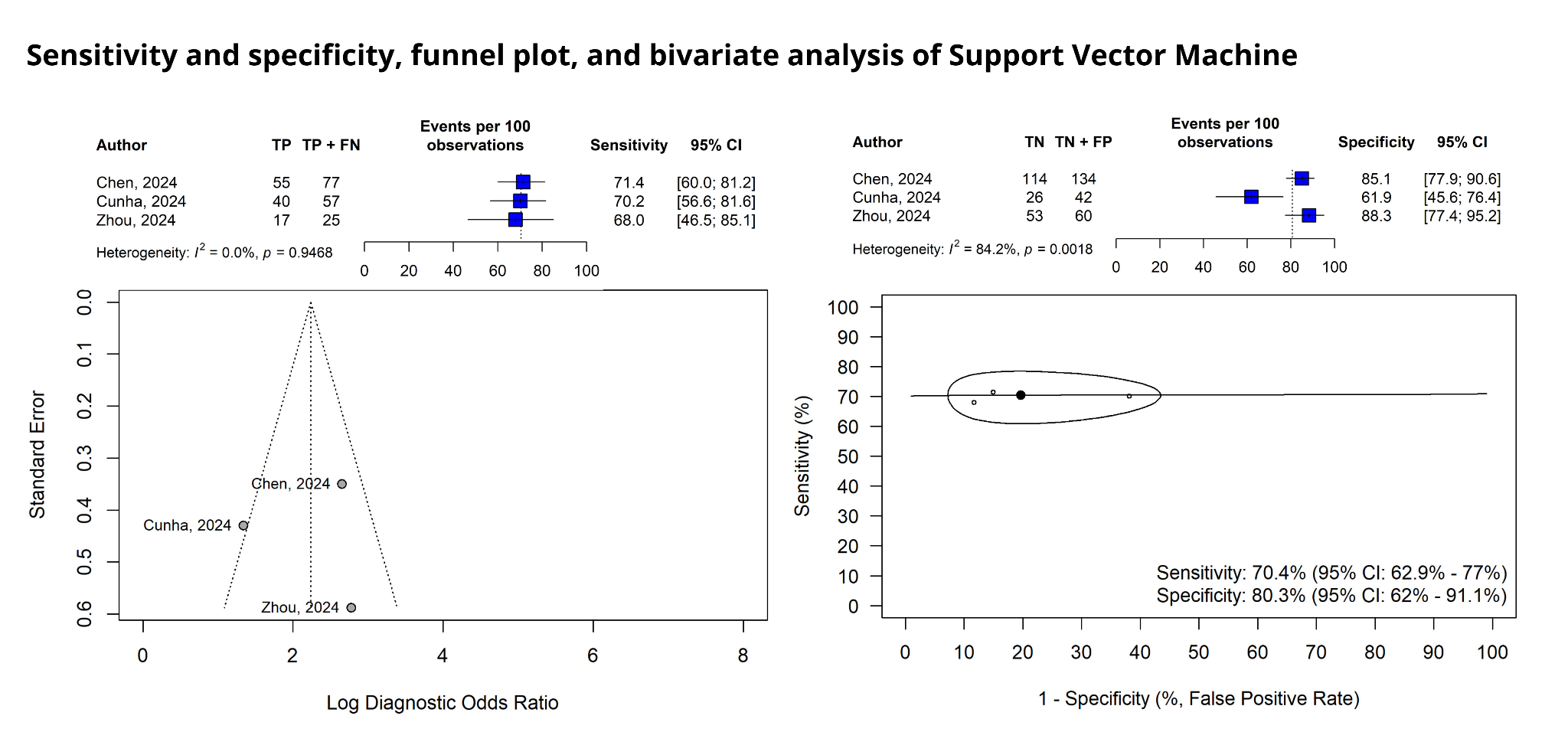


**Supplementary Figure 10** Sensitivity, specificity, funnel plot and bivariate analysis of Light Gradient Boosting.


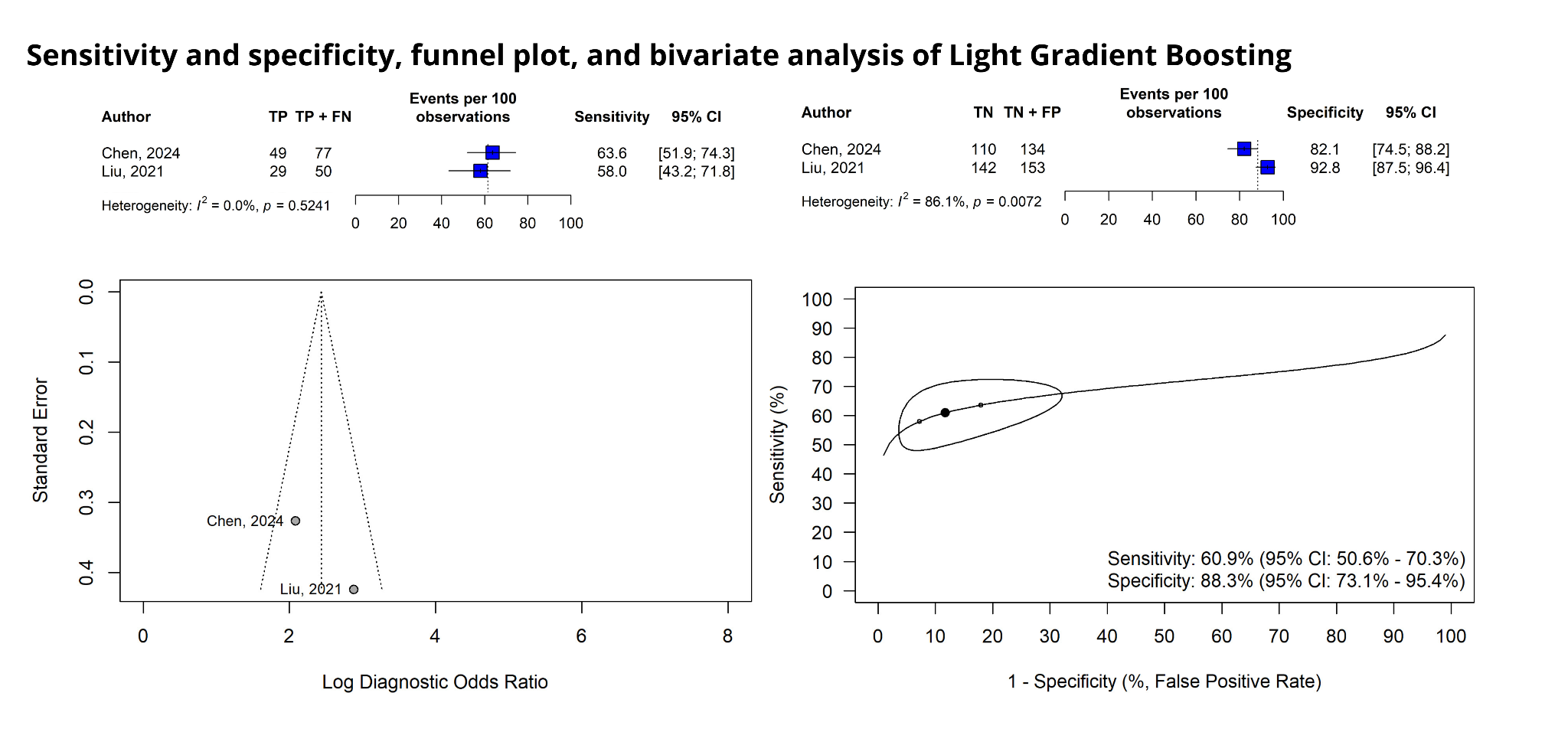


**Supplementary Figure 11** Sensitivity, specificity, funnel plot and bivariate analysis of Linear Discriminant Analysis.


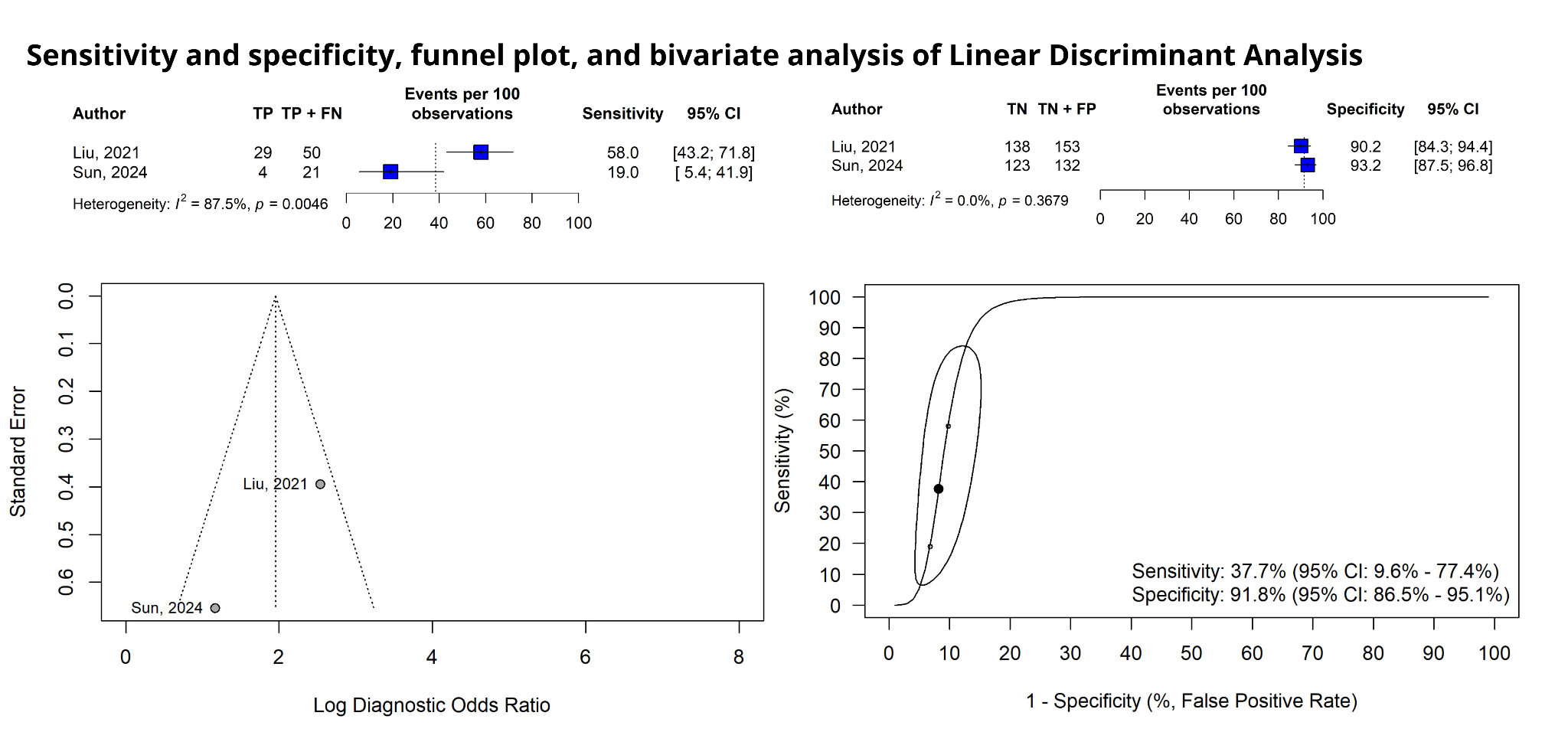

Supplement: Supplementary file 1 [file mmc1.docx]
